# Brazilian Society of Surgical Oncology: Guidelines for the Lymphadenectomy in Colorectal Cancer

**DOI:** 10.1002/jso.70000

**Published:** 2025-06-16

**Authors:** Jorge Mali, Marcus Valadão, Rodrigo Otavio Araujo, Carlos Bernardo Cola, Carlos Augusto Rodrigues Veo, Heládio Feitosa e Castro Neto, Alexandre Ferreira Oliveira, Reitan Ribeiro, Rodrigo Nascimento Pinheiro

**Affiliations:** ^1^ Department of Clinical Surgery State University of Londrina Londrina Brazil; ^2^ Division of Abdomino‐Pelvic Surgery Instituto Nacional de Cancer Rio de Janeiro Brazil; ^3^ Division of Clinical Research and Technological Development Instituto Nacional de Cancer Rio de Janeiro Brazil; ^4^ Department of Surgical Oncology Federal University of the State of Rio de Janeiro Rio de Janeiro Brazil; ^5^ Department of Colorectal Oncology Surgery Barretos Cancer Hospital Barretos Brazil; ^6^ Department of Surgical Oncology Cancer Institute of Ceara Ceara Brazil; ^7^ Department of Surgery Federal University of Juiz de Fora Juiz de Fora Brazil; ^8^ Department of Gynecology Oncology Erasto Gaertner Hospital Curitiba Brazil; ^9^ Surgical Oncology Service Base Hospital of Federal District Brasilia Brazil

**Keywords:** colorectal cancer, guideline, lymphadenectomy

## Abstract

The BSSO developed the present guidelines to provide recommendations based on current scientific evidence that is focused on the main topics related to the daily surgical practice of lymphadenectomy in colorectal cancer. Between October 2024 and February 2025, nine experts met to develop the guidelines for the lymphadenectomy in colorectal cancer. These guidelines summarize concisely the recommendations based on the most current scientific evidence on the most relevant aspects of the lymphadenectomy in colorectal cancer.

## 1 Introduction

Colorectal cancer is a public health problem in Brazil and worldwide, appearing as the main cause of death. According to Global Cancer Statistics 2022, more than 1.9 million new cases of colorectal cancer (including anal cancers) and 904 000 deaths were estimated to occur in 2022, representing close to one in 10 cancer cases and deaths. Overall, colorectal cancer ranks in third place in terms of incidence but second in terms of mortality [1]. In Brazil, for each year of the 2023–2025 triennium, approximately 21 970 cases of colon and rectal cancer are estimated in men and 23 660 in women. These values correspond to an estimated risk of 2078 new cases per 100 000 men and 2141 new cases per 100 000 women. Excluding nonmelanoma skin cancer, colon and rectal cancer is the third most common cancer type in Brazil [2]. The 5‐ year survival relative rate for colorectal cancer ranges from 91% for localized disease to 14% for distant disease [3]. Accurate tumor node metastasis (TNM) staging is essential for the treatment decisions and prognosis of colorectal cancer. Lymph nodes (LNs) are the most common sites of metastasis, and LN status is the most powerful prognostic factor [4]. Determining an adequate LN number may be challenging for pathologists as it requires thorough dissection to identify and quantify positive LNs, which significantly affect patient survival and prognosis [5].

## 2 Definition of the Problem

Surgical resection remains the most effective therapy for colorectal cancer, and lymphadenectomy is a decisive factor for the prognostic and therapeutic staging. The American Joint Committee on Cancer (AJCC) TNM system recommends retrieving a minimum of 12 LNs for accurate staging in colorectal cancer [6]. This recommendation has been included in various clinical practice guidelines, including those of the National Comprehensive Cancer Network (NCCN) and the European Society for Medical Oncology (ESMO) [7, 8, 9].

Considering the importance of lymphadenectomy in the surgical treatment of colorectal cancer, the Brazilian Society of Surgical Oncology (BSSO) brought together experts to create this guideline, based on the best scientific evidence available, with the aim of guiding the indication and extent of lymphadenectomy in colorectal cancer.

## 3 Methods

Between October 2024 and February 2025, nine experts in colorectal cancer surgery met to develop the guidelines for the lymphadenectomy in colorectal cancer. A total of 12 relevant topics were distributed among the experts. For the development of these guidelines, prospective randomized clinical trials and meta‐analyses were preferred. Direct searches of the references of the primary articles and practice guidelines or consensus documents of relevant societies, were also performed. The methodological quality of a final list with 127 sources was evaluated, all the evidence was examined and revised, and the treatment guideline was formulated by the nine‐expert committee. An adapted version of the Infectious Diseases Society of America‐United States Public Health Service Grading System was used to rank the level of evidence (Table 1) and grade of recommendation (Table 2). To reach a final consensus, all the topics were reviewed via a videoconference meeting that was attended by all nine of the experts.

**Table 1:** Levels of evidence.

Levels of evidence	
I	Evidence from at least one large randomized controlled trial with good methodological quality (low potential bias) or meta‐analyses of well‐conducted randomized trials without sample heterogeneity
II	Small randomized trials or large randomized trials with suspected bias (poor methodological quality), meta‐analyses of these trials, or trials with demonstrated sample heterogeneity
III	Prospective cohort studies
IV	Retrospective cohort or case‐control studies
V	Studies without control groups, case reports, and expert advice

**Table 2:** Grades of recommendation.

Grade of recommendation	
A	Strong evidence of efficacy with significant clinical benefit; strongly recommended
B	Strong or moderate evidence of efficacy but limited clinical benefit; usually recommended
C	Insufficient evidence of efficacy or benefit does not outweigh risk or disadvantages (ie, adverse events, costs, and other factors); recommended in some cases
D	Moderate evidence of ineffectiveness or occurrence of adverse outcomes; rarely recommended
E	Strong evidence of ineffectiveness or occurrence of adverse outcomes; never recommended

## 4 The Role of Lymphadenectomy in Colorectal Cancer

Lymphadenectomy in colorectal cancer plays an important role in staging and treatment, in addition to being considered as a major prognostic fator. LNs metastasis remains one the most important variable to select patients for neoadjuvant and/or adjuvant treatment. Lymphadenectomy in colorectal should be adequately performed and at least 12 LNs should be removed.

Level of evidence: II

Grade of recommendation: A

Surgical resection and regional lymphadenectomy remain the most effective treatment for colorectal cancer disease, and the extent of LN dissection plays a role in staging, treatment, prognosis, and directly influences survival [10, 11]. Tumor location, grade, age, tumor size, T stage, and carcinoembryonary antigen (CEA) are recognized as independent predictive factors for LNs metastasis in colorectal cancer patients. The number of LNs surgically removed is directly correlated with patient survival [12, 13, 14, 15]. Furthermore, the number of LNs retrieved has repeatedly been validated as a powerful prognostic tool in patients with colorectal cancer. In particular, the absolute number of positive nodes has been identified as a highly effective predictor of adverse outcome, as shown by worsening the prognosis with increasing number of LNs involved by cancer [16, 17].

The NCCN, the College of American Pathologists, and the AJCC suggest a minimum of 12 LNs to establish an accurate N stage [17, 18, 19]. Therefore, a lymphadenectomy can be considered adequate when at least 12 LNs are removed.

In colorectal cancer, an adequate staging, the decision to deliver adjuvant therapy, and patient survival are strongly influenced by meticulous lymphadenectomy [20, 21]. Previous studies have demonstrated that the number of LNs removed is positively associated with increased survival [12, 14, 21].

The regional lymphadenectomy and an adequate oncologic surgery, remains the fundamentals surgical principles in the resection for colorectal cancer and the main determinant of cancer outcomes. Moreover, the LNs dissection is an objective quantifiable marker that reflect the adequacy of surgical care in routine clinical practice [22, 23]. In 2007, after considering the recommendations of various professional's committees on colorectal cancer, the National Quality Forum endorsed the harvest of at least 12 LNs as the standard quality indicator and the way of improving survival. Importantly, LNs removed surgically as well as assessed pathologically governs not only the accuracy of tumor staging but also therapeutic decision‐making [24, 25].

Several studies have demonstrated that the analysis of a larger number of LNs results in a survival advantage for patients with stage II and III colorectal cancer [26, 27, 28, 29]. Lykke et al. demonstrated that in patients with more than 12 nodes retrieved, there was a significantly higher proportion of stage III disease, indicating that stage migration takes place when high numbers of LNs are harvested [27]. The LN ratio, defined as the number of positive LNs divided by the total number of retrieved nodes, has been proposed as a more reliable prognostic indicator than the absolute number of involved LNs [30, 31]. LN ratio was identified as an independent predictor of disease‐free survival, overall survival, and cancer‐specific survival in stage III colorectal cancer [30, 31, 32]. The LN ratio can be considered to identify patients at high risk of disease recurrence [33].

## 5 Colorectal LN Anatomy— Japanese Classification

**Figure 1:**
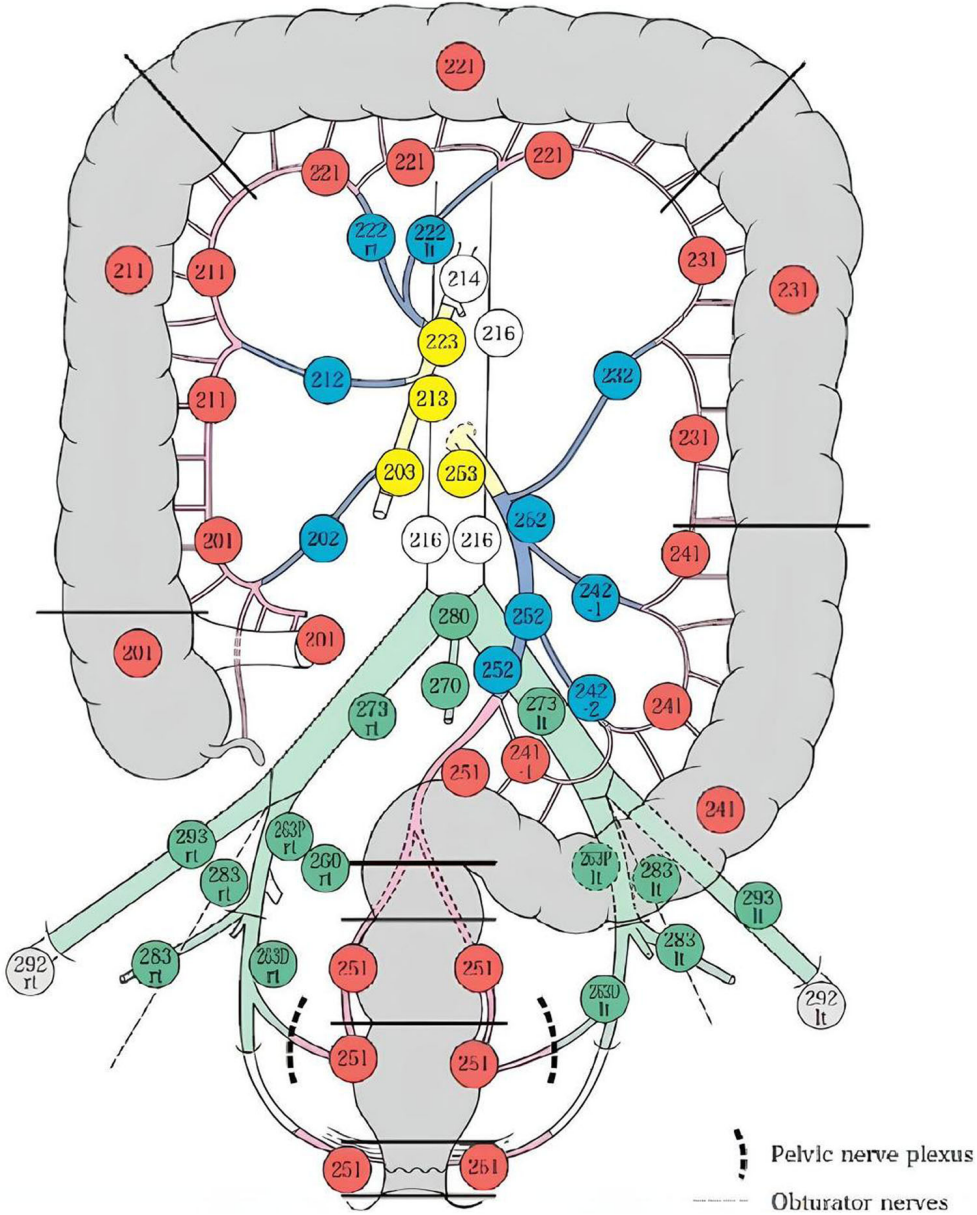
Lymph node groups and station numbers (Adaptaded from Hashiguchi et al. [10] and Kotake K. [35]). Red: Pericolic/perirectal lymph nodes. Blue: Intermediate lymph nodes. Yellow/Green: Main lymph nodes/Lateral lymph nodes. White: Lymph nodes proximal to the main lymph nodes. Grey: Others lymph nodes.

The BSSO uses the LN anatomical classification of the Japanese Society of Colorectal Cancer to define the extent of lymphadenectomy [34]. The LNs of the colon, rectum, and anus are classified and numbered according to their anatomy in relation to the arteries (Figure 1 and Table 3). The LNs subject to lymphadenectomy (regional LNs) consist of three groups: pericolic/perirectal, intermediate, and main LNs (Table 4). In addition, the lateral pelvic nodes are included as a fourth group in the rectum. The extent of lymphadenectomy of regional LNs can vary according to the anatomical location of the tumor in relation to its irrigating artery or arteries.

**Table 3:** Lymph nodes groups (Adaptaded from Hashiguchi et al. [10]).

	Superior mesenteric artery	Inferior mesenteric artery	Iliac artery
a Pericolic/perirectal lymph nodes	Lymph nodes along the marginal arteries and vasa recta of the colon (Pericolic nodes)	Lymph nodes along the marginal arteries and vasa recta of the colon (Pericolic nodes) Lymph nodes along the terminal sigmoid artery (Pericolic nodes) Lymph nodes in the mesorectum along the superior rectal artery (Perirectal nodes)	Lymph nodes medial to the pelvic nerve plexus along the middle rectal artery (Perirectal nodes)
b Intermediate lymph nodes	Lymph nodes along the colic arteries (Ileocolic nodes) (Right colic nodes) (Right middle colic nodes) (Left middle colic nodes)	Lymph nodes along the left colic and sigmoid arteries (Left colic nodes) (Sigmoid colic nodes) Lymph nodes along the inferior mesenteric artery between the origin of the left colic artery and the origin of the terminal sigmoid artery (Inferior mesenteric trunk nodes)	
c Main lymph nodes (Lateral lymph nodes)	Lymph nodes at the origin of each colic artery (Ileocolic root nodes) (Right colic root nodes) (Middle colic root nodes)	Lymph nodes along the inferior mesenteric artery proximal to the origin of the left colic artery (Inferior mesenteric trunk nodes)	Lymph nodes along the internal iliac arteries (Distal internal iliac nodes) (Proximal internal iliac nodes) Lymph nodes along the common iliac arteries (Common iliac nodes) Lymph nodes along the obturator vessels and nerves (Obturator nodes) Lymph nodes along the external iliac arteries (External iliac nodes)
d Lymph nodes proximal to the Main lymph nodes	Lymph nodes along the superior mesenteric artery (Superior mesenteric nodes) Lymph nodes around the abdominal aorta and the inferior vena cava (Para‐aortic nodes)	Lymph nodes around the abdominal aorta and the inferior vena cava (Para‐aortic nodes)	Lymph nodes around the abdominal aorta and the inferior vena cava (Para‐aortic nodes)
e Other lymph nodes	Infrapyloric lymph nodes (Infrapyloric nodes) Lymph nodes along the gastroepiploic vessels (Gastroepiploic nodes) Lymph nodes at splenic hilum (Splenic hilum nodes)		Lymph nodes around aortic bifurcation (Aortic bifurcation nodes) Presacral lymph nodes (Median sacral nodes) (Lateral sacral nodes) Lymph nodes in the inguinal area (Inguinal nodes)

*Note:* The significance of color in Table 3 corresponds to the colors of the Lymph nodes groups described in Figure 1.

**Table 4:** Lymph nodes station numbers (Adaptaded from Hashiguchi et al. [10]).

**Region**	**Pericolic/perirectal lymph nodes**	**Intermediate lymph nodes**	**Main lymph nodes (lateral lymph nodes)**	**Lymph nodes proximal to the main lymph nodes**	**Other lymph nodes**
Superior mesenteric artery					
Ileocolic arteries	201 (Pericolic nodes)	202 (Ileocolic nodes)	203 (Ileocolic root nodes)		
Right colic artery	211 (Pericolic nodes)	212 (Right colic nodes)	213 (Right colic root nodes)	214 (Superior mesenteric nodes)	
Right middle colic artery	221 (Pericolic nodes)	222‐rt (Right middle colic nodes)		216 (Para‐aortic nodes)	
Left middle colic artery	221 (Pericolic nodes)	222‐lt (Left middle colic nodes)	223 (Middle colic root nodes)		206 (Infrapyloric nodes)
Inferior mesenteric artery					204 (Gastroepiploic nodes)
Left colic artery	231 (Pericolic nodes)	232 (Left colic nodes)			210 (Splenic hilum nodes)
Sigmoid artery					
First	241‐1 (Pericolic nodes)	242‐1 (First sigmoid colic nodes)	253 (Inferior mesenteric nodes)	216 (Para‐aortic nodes)	
Second	241‐2 (Pericolic nodes)	242‐2 (Second sigmoid colic nodes)			
Terminal sigmoid artery	241‐t (Pericolic nodes)				
Superior rectal artery	251 (Perirectal nodes)	252 (Inferior mesenteric trunk nodes)			
Iliac artery					
Middle rectal artery	251 (Perirectal nodes)				
Internal iliac artery			263D (rt + lt) (Distal internal iliac nodes) 263P (rt + lt) (Proximal internal iliac nodes)		
Common iliac artery			273 (rt + lt) (Common iliac nodes)	216 (Para‐aortic nodes)	260 (rt + lt) (Lateral sacral nodes)
Obturator vessels			283 (rt + lt) (Obturator nodes)		270 (Median sacral nodes)
					280 (Aortic bifurcation nodes)
External iliac artery			293 (rt + lt) (External iliac nodes)		292 (rt + lt) (Inguinal nodes)

*Note:* The significance of color in Table 4 corresponds to lymph node station numbers described Figure 1.

## 6 Lymphadenectomy in Colon Cancer

D2 dissection should be the routine procedure in patients with colon cancer. A minimum of 12 LNs should be examined to adequately establish N stage.

Level of evidence: II

Grade of recommendation: A

Staging in colon cancer is based on the TNM system. In the 8th edition of the AJCC, regional LN classification includes N1a (1 positive LN); N1b (2–3 positive LNs); N2a (4–6 positive nodes); and N2b (7 or more positive nodes). In addition, tumor deposit(s) in the subserosa, mesentery, or non‐peritonealized pericolic or perirectal tissues without regional nodal metastasis (i.e., satellite tumor nodules) have been classified as N1c. Survival is inversely correlated with N stage (N0, N1a, N1b, N2a, and N2b) [36]. This guideline recommends examination of ≥ 12 LNs to adequately establish the N stage. This recommendation is supported by the 8th edition of the AJCC Cancer Staging ManuaL [36]. There is evidence suggesting that a greater number of nodes may need to be examined in some situations, particularly for T4 lesions, to provide an adequate assessment of disease stage [37]. Patients with < 12 nodes have been examined on the surgical specimen must be considered incompletely operated and suboptimally staged. This subgroup should be considered to be at higher risk, regardless of nodal status N0.

The extent of LN dissection to be performed during colon cancer surgery is determined by the preoperative clinical and imaging findings, and intraoperative assessment (Table 5). Standard D2 dissection should be the routine procedure in patients with colon cancer, regardless of tumor location. The Proper technique for lymphadenectomy requires proximal ligation of the associated vascular pedicle(s) with complete dissection of pericolic/perirectal and intermediate LNs, associated with en bloc removal of the colonic segment and its mesentery. Other LNs, outside the standard field of resection or those at the origin of the vessel feeding the tumor (apical LN), that are considered clinically suspicious should be biopsied or removed, if possible. Resection must be complete to be considered curative, and any positive LNs left behind indicate an incomplete (R2) resection.

**Table 5:** Extent of lymph node dissection (D).

DX	The extent of lymph node dissection cannot be assessed
D0	Incomplete dissection of pericolic/perirectal lymph nodes
D1	Complete dissection of pericolic/perirectal lymph nodes
D2	Complete dissection of pericolic/perirectal and intermediate lymph nodes
D3	Complete dissection of all regional lymph nodes

The extent of colectomy is determined by the tumor location and the relationship between the primary tumor and the arterial arcade, containing the regional LNs, that irrigates a given segment of the colon. The bowel longitudinal resection margin should be 10 cm because the occurrence of metastases in pericolic LNs at a distance of more than 10 cm from the tumor is infrequent [38].

## 7 Metastases in Distant LNs

Excision of metastases in distant LNs can be considered in select cases.

Level of evidence: IV

Grade of recommendation: C

LN metastasis beyond the regional LNs is classified as M1. Although clear scientific evidence is lacking, the excision of metastases in distant LNs can be considered in selected cases. Some case series have reported long‐term survival following the resection of metastatic para‐aortic LNs, particularly in cases of isolated metastatic disease to the para‐aortic LNs [39, 40, 41, 42, 43, 44, 45, 46].

## 8 The Role of D3 Lymphadenectomy and Complete Mesocolic Excision (CME) for Cancer of the Right Colon

The extent of lymphadenectomy in right colon cancer is still a controversial topic. D2 lymphadenectomy remains the standard of care for the average right colon cancer patient. D3 lymphadenectomy can be offered in reference centers with expertise as an option to address extended radicality and the possibility of an improvement in local control.

Level of evidence: II

Grade of recommendation: C

### 8.1 CME Should be Recommended as the Standard Technique in Right Colon Cancer Surgery

Level of evidence: II

Grade of recommendation: B

D2 lymphadenectomy remains the standard of care for the average right colon cancer patient but D3 lymphadenectomy can be offered in reference centers with expertise as an option to address extended radicality and the possibility of an improvement in local control. Although the subject is debatable, there is a theoretical rationale and substantial retrospective data addressing a benefit for the extended lymphadenectomy for right colon cancer, mostly derived from institutions with large volume and experience with this procedure. In these institutions, D3 lymphadenectomy associated with CME has been demonstrated to be safe and to increase surgical radicality considering parameters such as the number of resected LNs and the number of positive LNs, thus allowing for more accurate LN staging. However, survival data derived from the two prospective randomized studies published to this date showed conflicting results and do not support a definitive recommendation, and long‐term results from a third randomized trial are still awaited. D2 lymphadenectomy remains the standard of care for the average right colon cancer patient but CME can be offered in reference centers with expertise as an option to address extended radicality and the possibility of an improvement in local control.

The radical hemicolectomy technique involves ligation of the ileocolic vessels near their origin in the superior mesenteric artery (SMA) and superior mesenteric vein (SMV), and ligation of the right colic artery (RCA) when present or the right branch of the middle colic artery (MCA) in the absence of a proper RCA. However, some authors advocate a more radical approach for colon cancer based on the concept and on the excellent local control results obtained with the adoption of total mesorectal excision (TME), standardized by Professor Bill Heald 40 years ago. The adoption and methodization of the principles of TME for colon cancer was popularized by Hohenberger et al. from the University of Erlangen, establishing the principles of CME associated with central vascular ligation (CVL). The technique of CME comprises a complete surgical dissection of an intact mesocolic plane with transection of the feeding vessels at their origin and the removal of all regional lymphatic tissue. Using this technique, the authors demonstrated a significant reduction in the local recurrence rate from 6.5% to 3.6% [47]. West et al. from the United Kingdom and Germany compared surgical specimens from the University of Erlangen with those obtained in Leeds using the traditional or non‐standardized colonic resection technique, and concluded that surgeons in Erlangen who routinely performed CME and LVC surgery removed more mesocolon and were more likely to dissect in the mesocolic plane and remove an intact mesocolic fascia when compared to standard resections in Leeds, which could explain the higher survival rate of patients operated at that center in Germany compared to that center in England [48]. At the same time, in Japan, an extended D3 lymphadenectomy has been recommended for tumors staged as T2‐4, reserving D1 lymphadenectomy for only T1 tumors [35].

Although the CME adopted in Europe and D3 lymphadenectomy preconized in Japan both share some common features, they are not equal. These two terms (CME and D3 lymphadenectomy) have been used in the literature as sinonyms incorrectally, but they are not sinonyms [49]. CME refers to complete removal of the mesocolic envelope through dissection along the embryological plane between the visceral and parietal fascia. On the other hand, D3 lymphadenectomy in right colon cancer refers to CVL (high vascular ligation on the most proximal aspect of the feeding arteries and veins) associated with the removal of all nodal tissue along the SMV and lateral to the SMA.

#### 8.1.1 Surgical Technique

**Figure 2:**
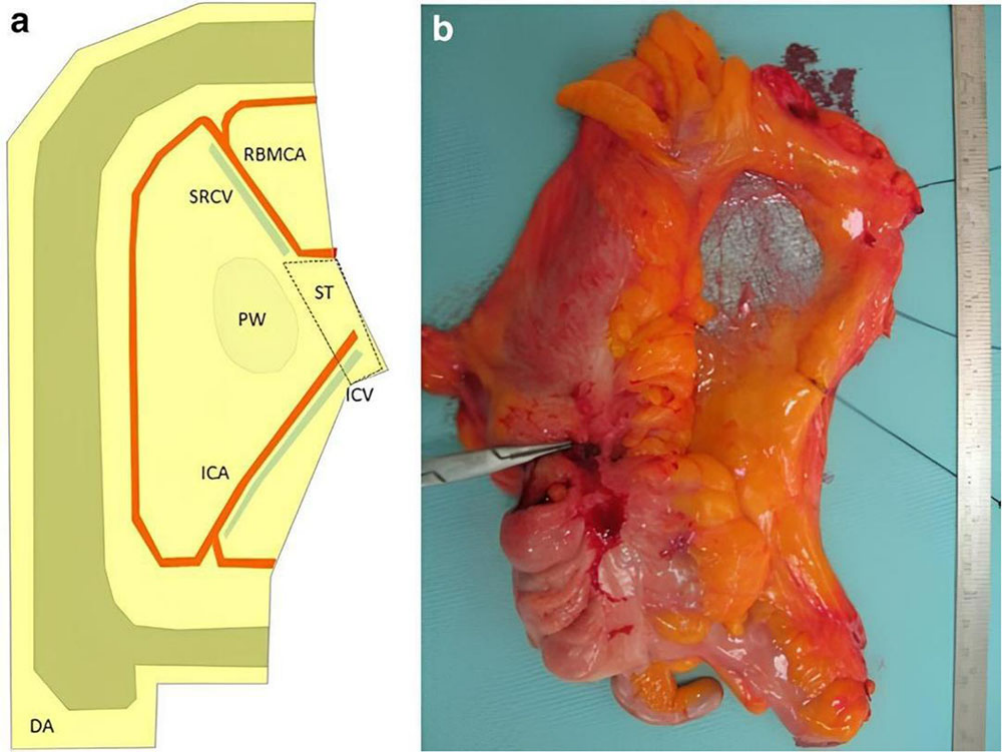
Schematic view (a) and typical example (b) of the Benz Type 0 specimen. DA, dissection area; ICA, ileocolic artery; ICV, ileocolic vein; PW, mesocolic peritoneal window; RBMCA, right branch of the middle colic artery; SRCV, superior right colic vein; ST, surgical trunk. Figure adapted from Benz et al. [51].

In the CME technique for right and transverse colon cancer, the ileocolic vessels are divided after complete mobilization of the mesocolon. Next, the right colic vessels are ligated, if present. In cancer of the cecum and ascending colon, the right branch of the middle colic vessels are also tied and divided. However, the middle colic vessels must be divided at their root for tumors located in the hepatic flexure and in the proximal transverse colon. According to the Japanese guideline, resection of a free colon margin of 10 cm is recommended to include the pericolic LNs because in a recent Japanese study including around 3000 patients undergoing colectomy (70% of which were laparoscopic), 99.9% of metastases in pericolic LNs were located within 10 cm of the primary tumor [50]. Additionally, Benz et al. in 2019 established a criteria for classifying the anatomopathological specimen concerning radicality into four stages for tumors of the right colon (Benz 0−III) [51], an evolution of West's original classification [52]. Resection specimens would be considered CME only if Benz 0, that is, the ties of the ileocolic vessels and the middle colic vessels should be connected by a tissue called “surgical trunk,” the lymphatic tissue bridge that covers the SMV between Ileo‐colic artery (ICA) and the right branch of the middle colic artery (RBMCA). The mesocolic peritoneal window, that is, the mesocolon fascial tissue between the trunk of the ileocolic vessels (ICA and ICV) and the RBMCA, has a completely intact medial covering of mesocolic tissue (Figure 2).

Clinical evidence of the technique:

The current JSCCR guidelines are based on registry data comprising 25 617 patients treated for colorectal adenocarcinoma between 2000 and 2004. Of these patients, 15 360 patients had colon cancer; this cohort demonstrated a 0.6% risk of N3 nodal metastases in pT2 colon tumors (corresponding to a D3 area of dissection), which increased to 5.5% in pT4b tumors [35]. These data suggest a benefit for routine D3 Lymphadenectomy surgery in all but stage I colon cancer cases.

Among nine meta‐analyses published until 2022 comparing CME/D3 lymphadenectomy to conventional surgery, seven of these demonstrated a benefit in overall survival at 5 years and five meta‐analyses demonstrated a benefit in disease‐free survival [53]. However, to date, the existing evidence is observational or retrospective and only three randomized clinical trials comparing CME/D3 lymphadenectomy to conventional colectomy have results published, as follows:

The Russian COLD study (Study of Oncological Outcomes of D3 Lymph Node Dissection in Colon Cancer) presented the short‐term results of 100 patients and demonstrated a significantly higher rate of nodal positivity (46% vs. 26%) comparing resections with D3 lymphadenectomy versus D2, despite there was no difference between the average number of LNs resected using the two techniques (28 vs. 27) [53]. Data from long‐term oncological control and survival results are pending.

In a study conducted at a single Chinese institution (Department of Colorectal Surgery, Gansu Provincial Hospital, Lanzhou, China), Yan et al. randomized 196 stage III colon patients to laparoscopic CME/D3 lymphadenectomy or traditional surgery. This study demonstrated a significant increase in the number of resected LNs in the group undergoing CME/D3 lymphadenectomy (25 vs*.* 17), as well as in the proportion of positive LNs [54]. Furthermore, the group undergoing laparoscopic CME/D3 lymphadenectomy had less blood loss (129 vs. 162 mL) and shorter hospital stay (13 vs. 15 days). Finally, in this study, the group undergoing CME/D3 lymphadenectomy demonstrated a significantly greater increase in overall survival rates (81.6% [80/96] and 77.1% [74/96]) and disease‐free survival (74.5% [70/94] vs. 63.8% [60/94]) after a follow‐up period of more than 40 months in each arm.

Finally, in a third study, a large phase 3 multicenter study from China—RELARC, 1072 patients from 17 hospitals were randomized to compare laparoscopic CME/D3 lymphadenectomy versus D2 colectomy [55]. Pathological results significantly favored CME/D3 lymphadenectomy in terms of area of resected mesocolon in the specimen, quality of CME, and the number of LNs resected (26 vs. 23). Three‐year survival data from the RELARC study was recently published [56]. The authors compared 495 patients submitted to CME/D3 lymphadenectomy to 500 patients submitted to standard D2 lymphadenectomy between 2019 and 2019 and found no significant difference between the groups in 3‐year DFS (86.1% in the CME group vs. 81.9% in the D2 group, *p* = 0.06) or in 3‐year OS (94.7% in the CME group vs. 92.6% in D2 group, *p* = 0.17), concluding that the addition of CME/D3 lymphadenectomy (with dissection of the the central lymphatic tissue covering the surface of the SMA and SMV and the root of the RCA) offered no benefit to the standard D2 dissection. Interestingly, in this study the control arm with D2 lymphadenectomy was quite radical and also included ligature of the colic feeding vessels at the right edge of the SMV and the pericolic and all intermediate LNs were removed, which can explain the similarity in outcomes between the two groups) [56]. In summary, although the subject is debatable, there is a theoretical rationale and substantial retrospective data addressing a benefit for the extended lymphadenectomy for right colon cancer, mostly derived from institutions with large volume and experience with this procedure. In these institutions, CME/D3 lymphadenectomy has been demonstrated to be safe and to increase surgical radicality considering parameters such as the number of resected LNs and the number of positive LNs, thus allowing for more accurate LN staging. However, survival data derived from the two prospective randomized studies published to this date showed conflicting results and do not support a definitive recommendation, and long‐term results from a third randomized trial are still awaited.

## 9 Preservation of the Left Colic Artery (LCA) in Sigmoid and Rectal Cancer Surgery

Preservation of the LCA in sigmoid and rectal cancer surgery appears to be associated with a lower risk of anastomotic leakage (AL).

Level of evidence: II

Grade of recommendation: B

If low ligation (preservation of the colic artery) is performed, lymphadenectomy of the IMA (inferior mesenteric artery) root (apical LN dissection) should be recommended to guarantee adequate lymphatic clearance.

Level of evidence: II

Grade of recommendation: B

The standard operative procedure for curative resection of intraperitoneal rectal cancer and sigmoid colon cancer includes removal of the tumor, wide resection of the colonic mesentery, and ligation of inferior mesenteric vessels [57]. There are two options for ligation of the IMA: (1) High ligation of IMA (non‐preservation of LCA with ligation of IMA at the aortic origin) and (2) Low ligation of IMA (preservation of LCA with ligation of IMA below the origin of LCA). High‐tie ligation has been advocated because it allows more radical resection and more accurate pathological staging [58, 59, 60, 61, 62, 63]. Others favor low‐tie ligation because of increased blood flow to the proximal end of the anastomosis and the potential reduction in AL rate [64, 65, 66, 67, 68, 69, 70]. This debate goes all the way back to the descriptions by Miles [71] and Moynihan [72] in 1908 and until nowadays, there is no consensus on where to divide the IMA. Several reviews [73, 74, 75, 76] found no significant difference between high and low tie regarding short‐ and long‐term outcomes, with different authors recommending different methods based on non‐randomized clinical trials.

Several studies have shown that colonic blood flow (colonic perfusion was measured with laser Doppler flowmetry) is decreased in high‐tie compared with low‐tie ligation [68, 69, 70]. These findings suggest that anastomoses may benefit from better perfusion when low‐tie ligation is performed. Interestingly, the initial meta‐analyses have arrived at different conclusions about the incidence of AL with the two approaches. Fan et al. [77] reported that low ligation of the IMA may lower the risk of AL, whereas pooled data from studies of Cirocchi et al. [76] and Yang et al. [78] showed that there was no significant difference in the incidence of AL with the two approaches. However, two most recent meta‐analysis showed that the preservation of LCA was associated with significantly less AL [78, 79]. Zeng et al. [79] included 18 articles (14 non‐randomized and 4 randomized clinical trials) and 5917 patients (3652 patients underwent low ligation and 2265 patients underwent high ligation of the IMA) and reported the following results: AL was 9.8% in high ligation patients versus 7.0% in low ligation patients; the risk of AL was significantly higher in high ligation patients (odds ratio [OR] = 1.33; 95% confidence interval [CI] 1.10−1.62; *p* = 0.004); overall morbidity was also significantly higher in high ligation patients (OR = 1.39; 95% CI, 1.05‐1.68; *p* = 0.05); and postoperative mortality, number of harvested LNs, overall recurrence rate, and 5‐year survival rate did not differ significantly between the two groups. Yang et al. [80] included 24 articles (20 non‐randomized and 4 randomized clinical trials) and 8456 patients (4058 patients underwent high ligation and 4398 patients underwent low ligation of the IMA with LCA preservation). They found that the preservation of LCA was associated with significantly less AL (OR 1.23, 95% CI 1.02−1.48, *p* = 0.03) and there were no significant differences between the two groups in terms of sexual dysfunction, urinary retention, the number of apical nodes, and long‐term oncologic outcomes.

## 10 Lymphadenectomy in Rectal Cancer

Standard lymphadenectomy in rectal cancer should systematically involve the LNs of the regional drainage chains, that is, the perirectal LNs contained in the mesorectum, along the superior rectal artery and IMA trunk, regardless of the level of IMA ligation. A minimum of 12 LNs should be examined to adequately establish the N stage.

Level of evidence: III

Grade of recommendation: A

Before diving into the current lymphadenectomy standards for rectal cancer, the definition of the proper rectum is an important first step, as it defines the surgical treatment and the lymphadenectomy's adequate extent. The most simple rectal anatomic division is comprised of the extraperitoneal lower 2/3 rectum (*proper rectum*), in which lower end we find the anal canal's top, and the intraperitoneal upper 1/3, in which upper end we find the retosigmoidean junction (at an average of 15 cm from the anal verge). The lower third's lymphatic drainage is primarily cephalic toward the upper rectal pedicle and inferior mesenteric root, but there is also lateral drainage along the middle rectal vessels to internal iliac LNs [81, 82, 83].

In this topic, we will focus on the *proper rectum's* lymphadenectomy, which is subdivided in middle and lower rectum.

The standard and minimal radical surgical treatment of the middle and lower rectal cancer involves a complete TME, following doctor Heald's holly plane of the *proper rectum fascia* with ligation of the IMA at its origin (D3 lymphadenetcomy) [82]. The preservation or sacrifice of the LCA is discussed elsewhere in this guideline.

By definition, a D2 lymphadenectomy would include the removal of the perirectal and intermediate LNs, while a D3 lymphadenectomy would also involve those located in the trunk of the IMA [84]. Regional LN involvement is one of the main prognostic factors for the progression of rectal cancer, as well as the ratio between the resected and positive LNs [85, 86]. However, there are divergent opinions about what constitutes an adequate lymphadenectomy, necessary for curative intent. By consensus, it should include the analysis of 12 or more regional LNs, although this number may be lower in patients undergoing neoadjuvant treatment and may also vary depending on other factors, such as age, sex, degrees of differentiation, and tumor sites [87, 88]. The average number of LNs obtained after a neoadjuvant surgery is significantly lower [89, 90].

Although there are controversies regarding the ideal level in different guidelines, evidence of the need for a systematic lymphadenectomy is consistent. Our recommendation is that a D3 lymphadenectomy should be performed systematically in cases where a standard lymphadenectomy is indicated, regardless of the level of IMA ligation.

## 11 Lateral Pelvic Lymphadenectomy (LPL)

A LPL is still a controversial topic. In the absence of suspicious LNs, a LPL is not recommended routinely. A LPL (on the affected side) is recommended in the presence of suspicious LNs.

Level of evidence: II

Grade of recommendation: B

The incidence of LPLN metastasis in locally advanced rectal cancer fluctuates from 7% to 24%, and the outcome of cases with LPLN metastasis is poor [91, 92]. International consensus about the management of LPLN remains controversial and the additional value of lateral lymph node dissection (LLND) in reducing LLR is confounded by different approach between Eastern and Western countries [93].

According to the Japanese Society for Cancer of the Colon and Rectum (JSCCR) guidelines for the treatment of colorectal cancer, LPLN metastasis is considered regional disease and controlled by LPLD, and TME plus LPLD is the standard surgical procedure when the lower border of the tumor is located distal to the peritoneal reflection and the tumor has invaded beyond the muscularis propria [94, 95, 96]. Prophylactic Lateral LN dissection is recommended, even if lateral LN metastasis is not detected by a preoperative diagnosis. This approach has been supported by reduction the incidence of locorregional recurrence from 12.6% to 7.4%, with acceptable morbidity. In contrast, western countries have traditionally performed neoadjuvant chemoradiotherapy (NCRT) and TME for all patients with stage II/III extraperitoneal rectal cancer. The addition of NCRT has reduced the local recurrence from 11.3% to 5.8%. The NCCN guidelines recommend TME after CRT for stage II‐III rectal cancer [97]. NCCN guideline does not recommended prophylactic dissection unless metastasis is clinically suspicious. The ESMO guidelines also do not particularly recommend prophylactic dissection for LPLNs without suspected metastasis, and declare that the addition of neoadjuvant CRT is considered superior (higher efficacy and/or less morbidity) to surgical resection of the LLN, but they do mention that this conclusion is not based on high‐quality research [98]. The American Society of colon and rectal surgeons also strongly recomends not to perform an LLND in the absence of enlarged LLN, but they do not mention how to treat patients with enlarged LLN [99]. Western surgeons believe that the lateral compartment can be adequately sterilized by neoadjuvant chemorradiation.

The JCOG0212 study aimed to confirm the non‐inferiority of the TME alone to the TME with LLND with the primary endpoints of relapse‐free survival [94]. This study was conducted for patients with a stage II/III rectal tumor below the peritoneal reflection and without enlarged LLN ( < 10 mm). The results could not confirm the non‐inferiority of TME alone. The frequency of local recurrence in the TME + LLND group was significantly lower than that in the TME alone group (7.4% vs. 12.6%). On the other hand, the relapse free survival curves of the two groups were very similar, and there was no significant difference in either the overall survival rate or local recurrence‐free survival rate as a secondary endpoint. The authors concluded that their findings supported the efficacy of TME with LLND in the setting without neoadjuvant therapy. The 7‐years analyses still failed to show a significant difference between TME with LLND and TME alone. In this study, only 7.3% of the patients in the TME + LLND group had positive pathological LLNs. These results indicate that prophylactic LLNDs in patients without enlarged LLNs might result in an overtreatment of this patient population. The authors also demonstrated that the short‐axis diameter ( > 5 mm) of the LLN is a predictive factor for pathological positivity. A subanalysis of the JCOG0212, only including patients with an LLN between 7 and 10 mm showed to have a lower lateral local recurrence‐free survival rate than patients with an LLN smaller than 7 mm (70.0% vs. 85.1%) [100, 101]. The relatively high LLR rate indicates that an LLND alone might not be sufficient in patients with an LLN ≥ 7 mm. The authors suggested that these high‐risk patients might benefit from a combined approach with CRT + TME + LLND [100, 101].

Recently, a multicentre retrospective study by Ogura et al. found that nodes along the internal iliac artery were less responsive to chemoradiation, and concluded that nodes measuring 7 mm or more on pre‐treatment MRI were predictors of lateral local recurrence [102]. The study reported 5‐year LLR rates of 52.3% following NCRT and TME surgery but without PLND. When PLND was performed, the 5‐year LLR risk was significantly reduced to 8.7% (*p* = 0.007).

Therefore, the optimal management of metastatic LPLN appears to be shifting toward a selective multimodal approach, with selective PLND post neoadjuvant therapy appearing to offer lower rates of local recurrence in several studies. Akiyoshi et al. performed a study with 127 patients with stage II‐III rectal cancer below the peritoneal reflection. PLND was performed in patients with suspicious LLN on pre‐neoadjuvant CT or MRI, using SAD (short‐axis diameter) criteria of ≥ 7 mm [103]. Patients with clinically enlarged LLN underwent PLND irrespective of findings on posttreatment restaging. The study observed that the recurrence rate did not differ between the two groups (2.7% for the CRT + TME + LLND group and 7.1% for the CRT + TME group; *p* = 0.27). Despite the worse oncological outcomes expected in patients with LLN metastasis, these patients have similar local recurrence rate compared with the non‐LLN group in case an LLND is performed. Ishihara et al. reported similar findings. PLND was again performed based on the presence of suspicious pre‐neoadjuvant nodes [104]. The study reported LLR rates of 0% and 0.9% in patients who underwent TME with PLND and TME only respectively, suggesting that the selective addition of PLND is key in achieving local control in the lateral pelvis. Kroon et al. investigated the added value of LLND after (C)RT in Western patients [105]. This study was conducted in six international referral centers from the Netherlands, Australia and the United States. Patients with an LLN short‐axis diameter ≥ 5 mm from MD Anderson Cancer Center in Houston, Texas, USA, underwent (C)RT + TME + LLND and were compared to patients from the other Western centers who were treated with only (C) RT + TME. The LLR rate was 3% for the LLND group and 11% for the non‐LLND group. This infers that Western patients with enlarged LLNs may also experience improved oncological outcomes after LLND in addition to (C)RT and TME. Ogura et al. [102] demonstrated, in a recent multicenter study with 1216 patients from the lateral node consortium, that neoadjuvant treatment and TME surgery is not always sufficient, as patients with primarily enlarged LNs (short‐axis diameter ≥ 7 mm) had a 19.5% risk of developing an LLR. This implies that neoadjuvant chemoradiation alone does not always sterilize the lateral compartments sufficiently. When an LLND was performed in patients with an LLN ≥ 7 mm, the risk of LLR declined to 5.7%, with pathological positive LLNs in 24.6% of the patients.

Several studies have attempted to retrospectively identify certain suspicious nodal characteristics on preoperative imaging, such as nodal size, appearance, and size reduction following neoadjuvant therapy [106, 107, 108, 109]. The size of an LPLN by magnetic resonance imaging (MRI) seems to be a better predictor for LLR than other characteristics such as irregular border and internal heterogeneity are not so accurate predictors of metastatic LLN [106, 107, 108, 109].

The boundaries of the lateral pelvic wall form a triangle between the external iliac artery laterally, ureter medially and urinary bladder caudally. Within this lie the internal iliac and obturador node groups, which can be dissected separately. All nerves and vessels are preserved unless invaded by metastatic node. The LLND can be performed by open, laparoscopic or robotic surgery. Multiple studies have investigated and compared the safety and oncological outcomes of the different approaches [109, 110, 111, 112, 113]. The results from these studies indicate that both laparoscopic and robotic LLND appear to have favorable short outcomes, especially considering the minimal amount of blood loss. Advantages of laparoscopic and robotic LLND might be a better surgical view in the deep pelvis, which can help with an increased accuracy during identification of the pelvic vessels and nerves. LLND can be a challenging procedure with risk of sexual and/or urinary disfunction. A meta‐analysis comparing TME + LLND with TME alone indicates worse functional outcomes after TME + LLND [114]. It is therefore important to carefully select the patients who might benefit from this procedure.

In conclusion, contemporary data appears to suggest that eastern and western treatment paradigms for lateral LNs in rectal cancer are slowly changing toward selective LLND.

The size of the LLN should not only be assessed on the pre‐neoadjuvant MRI but also the size on the restaging MRI should be used in the patient selection who can benefit from an LLND. LLND can be a challenging procedure with risk of intraoperative bleeding and sexual or urinary disfunction. Laparoscopic and robotic LLND procedures are techniques preferable over open surgery and result in less blood loss. It is essential that the obturator and internal iliac compartments should be resected in rectal cancer with enlarged LLN. Until more robust data is made available, a prudent choice would be to use not only MRI LN morphology but also SAD (short‐axis diameter) ≥ 7 mm on pre‐neoadjuvant MRI or SAD ≥ 5 mm after NCRT on the restaging MRI, as criterias for selective PLND.

## 12 Inguinal Lymphadenectomy

Inguinal lymph nodes (ILN) metastases from rectal adenocarcinoma can be considered for management. The combination of chemoradiotherapy and inguinal lymphadenectomy is recommended in the setting of resectable metastatic disease. If R0 resection can be achieved, inguinal lymphadenectomy must be considered.

Level of evidence: II

Grade of recommendation: B

The surgical treatment of inguinal lymph nodes metastasis (ILNM) secondary to rectal adenocarcinoma remains controversial. ILNM are a rare site of metastasis in rectal adenocarcinoma. Lymphatic drainage of the rectum is primarily via the mesorectal nodes and subsequently via the mesenteric vessels [115]. However, in low rectal cancers, lymphatic spread has been demonstrated via inguinal nodes in a manner similar to anal canal squamous cell cancers [116]. Therefore, there are two lymphatic routes from the rectum to inguinal LNs; one is a direct route, and the other is an indirect route which passes through internal and external iliac vessels.

The AJCC Cancer Staging Manual in the TNM 8th edition classification considers ILNM from rectal adenocarcinoma as a systemic disease (M1) [117], opposed to squamous cell carcinoma that is considered locoregional disease [118]. Whether ILNM should be treated with palliative or curative intent is unclear. Recent case series demonstrate significantly better survival outcomes for patients with ILNM treated with curative intent [119, 120].

There is limited evidence available about the treatment of patients with ILNM from rectal cancer. No guideline or consensus exists for management of ILNM from rectal adenocarcinoma. This guideline aimed to summarize all primary research involving ILNM from rectal adenocarcinoma to provide clinicians for optimal management strategies.

Recent studies have reported improved outcomes and acceptable survival for rectal adenocarcinoma with ILNM, particularly for isolated unilateral metastasis [121, 122, 123]. Wyatt et al. in systematic review demonstrates that in patients with isolated ILNM, modern chemoradiotherapy regimens in combination with surgical ILN dissection can achieve 5‐year overall survival rates of 53%−78%, significantly above that expected for rectal cancers with distant solid organ metastases [123]. The referral centers that demonstrate the best long‐term oncological outcomes utilize a combination of neoadjuvant chemoradiation and surgical ILN dissection.

### 12.1 Surgical Technique

At 2−3 cm below the inguinal ligament, a incision is made parallel to the inguinal ligament. The limits surrounding dissection are the inguinal ligament, adductor muscle, sartorius muscle, and intersection point between these muscle called the femoral Apex. Nowadays, it is unclear whether dissection should be limited to clinically positive nodes or more extensive nodal clearance of the inguinal region. Therefore, the optimal depth of dissection remains uncertain. Abd El Aziz et al. demonstrated no ipsilateral inguinal recurrence after limited dissection of just clinically positive nodes [120]. Hasegawa et al. suggest the additional excision of deep inguinal nodes [121]. Hagemans et al. recommended limiting dissection to superficial LNs only [122]. Both techniques have therefore been utilized and demonstrated success, but recommendations are not possible with small case series. Both superificial and deep inguinal LNs, including Cloquet's nodes, can be dissected. However, the optimal depth of dissection remains uncertain.

Preoperative diagnosis of ILNM is usually made with computed tomography, magnetic ressonance and/or fluorodeoxyglucose pósitron emission tomography (FDG‐PET). The most common definition of clinically suspect or positive ILN is abnormal morphology, short‐axis diamter ≥ 10 mm or FDG‐PET positive. No clear consensus about a biopsy of inguinal LN before inguinal node dissection.

Surgical treatment of ILNM from rectal adenocarcinoma may result in prolonged survival and possibly cure. Inguinal LN metastases should not be considered as an incurable disease, especially in patients with primary rectal cancer and solitary ILNM. Based on the acceptable prognosis [124] of patients with inguinal metastasis from rectal adenocarcinoma, inguinal LN dissection with curative intent can be considered in the surgical treatment combined with systemic therapy [125] in select patients if R0 resection is achieved. Prophylactic inguinal LN dissection should not be performed without a clinically positive ILN.

ILN dissection is associated with significant morbidity for patients. Wound complications such as infection, seroma, wound necrosis, and lymphorrhoea occur in more than 50% of patients after ILN dissection for other conditions such as melanoma, where lymphadenectomy is performed more routinely [126, 127]. As such, ILN dissection should be employed with caution.

Chemoradiotherapy appears to play an essential role. In two separate series reporting radiologically positive ILNM after NACR, just 4/15 (27%) and 9/13 (69%) had confirmed metastases on pathological examination, respectively [120, 121]. The limited data show that inguinal nodes are likely responsive to systemic chemotherapy and radiotherapy.

## Synopsis

This article reports the guidelines of lymphadenectomy in colorectal cancer of the Brazilian Society of Surgical Oncology. These recommendations are based on the best evidence available in the literature, and aim to guide the oncological surgeon make the best therapeutic decision.

## Data Availability

The authors have nothing to report.
